# Fruit and Vegetable By-Products to Fortify Spreadable Cheese

**DOI:** 10.3390/antiox7050061

**Published:** 2018-04-25

**Authors:** Annalisa Lucera, Cristina Costa, Valeria Marinelli, Maria Antonietta Saccotelli, Matteo Alessandro Del Nobile, Amalia Conte

**Affiliations:** Department of Agricultural Sciences, Food and Environment, University of Foggia, Via Napoli, 25–71121 Foggia, Italy; annalisa.lucera@unifg.it (A.L.); cristina.costa@unifg.it (C.C.); valeria.marinelli@unifg.it (V.M.); mariantoniettasaccotelli@gmail.com (M.A.S.); amalia.conte@unifg.it (A.C.)

**Keywords:** by-products, bioactive compounds, spreadable cheese, functional food, waste reduction

## Abstract

In this work, spreadable cheese was enriched with flours from by-products (red and white grape pomace, tomato peel, broccoli, corn bran, and artichokes) as sources of fibres and antioxidant compounds. The physicochemical and the sensory properties of all the cheese samples were analysed. Results revealed that total phenolic content, flavonoids, and antioxidant activity of samples containing grape pomace significantly increased, followed by broccoli, artichoke, corn bran, and tomato peel by-products, compared to the control cheese. Specifically, cheeses containing white and red grape pomace recorded high phenolic content (2.74 ± 0.04 and 2.34 ± 0.15 mg GAEs/g dw, respectively) compared to the control (0.66 mg GAEs/g dw).

## 1. Introduction

Nowadays, about one-third of fruit and vegetables in the form of peels, pips, and skins can be discarded during preparation and processing, thus producing significant wastes that represent one of the major problems of the food industry. Fruit and vegetable by-products can also be promising sources of functional compounds, being rich in carotenoids, dietary fibres, polyphenols, tocopherols, vitamins, and other substances [1]. For this reason, several works deal with the use of vegetable by-products to enhance the nutritional value or the technological properties of food. For example, mango peel powders were used to enhance the antioxidant properties of macaroni [2]. Sudha et al. [3] reported that the incorporation of apple pomace into wheat flour as a fibre source improved the rheological characteristics of cake. Grape marc extract was used in substitution of water to produce spaghetti, obtaining a food characterized by high content of phenolic compounds, flavonoids, and consequently high antioxidant activity [4]. Boubaker et al. [5] used artichoke stem powder as a source of dietary fibre and polyphenols to partially substitute wheat flour during bread making. Moreover, broccoli by-products were added in gluten-free mini sponge cakes to increase the polyphenolic and glucosinolates content [6].

In the dairy industry, there has been an increasing demand for healthy cheese, even though there are still very few studies available dealing with the use of fruit and vegetable by-products to fortify dairy products. Some valid examples about the use of wine grape pomace as antioxidant dietary fibre to enhance the nutritional value of yogurt and ice cream are reported [7,8]. Bertolino et al. [9] also demonstrated that the addition of hazelnut skins improved functional properties of yogurt. Marchiani et al. [10] assessed the physicochemical and nutritional quality of grape pomace powder to fortify semi-hard cheese. Abd Elhamid [11] proposed the incorporation of wheat bran in Kariesh cheese.

In this context, by-products from broccoli, artichoke, tomato peel, red and white grape pomace, and corn bran are taken into account, being interesting sources of healthy compounds (anthocyanins, catechins, flavonols, fibres, and phenolic acids) for spreadable cheese fortification [1]. This cheese is a soft, mild cheese, with slightly acidic tasting and diacetyl flavour. It is usually manufactured by the coagulation of milk mixed with cream by acidification with starter culture. It is used as spread on bagels, as salad dressing, and as ingredient for dessert. Generally, it is not a significant source of polyphenols and dietary fibres.

The objective of the present study was to evaluate the influence of the supplementation of different by-products (i.e., broccoli, artichoke, tomato peel, red and white grape pomaces by-products, and corn bran) on physicochemical and sensory quality of spreadable cheese. Cheese moisture content, pH, total phenolic compounds, total flavonoids, antioxidant activity, and sensory characterization were carried out for each cheese sample.

## 2. Materials and Methods

### 2.1. Raw Materials

White and red grape pomace (made up of skins, seeds, and stalks), tomato peels, broccoli stems and leaves, and artichoke external leaves were provided by Foggia, a local company (Southern Italy). The samples were dried at 30–35 °C in a dryer (SG600, Namad, Rome, Italy) for 48 h. The dried vegetables were reduced to fine powder by a hammer mill (16/BV-Beccaria s.r.l., Cuneo, Italy) and then stored at 4 °C until further utilization. The commercial corn bran was purchased from Limagrain Cereales Ingredients (Ennezat, France).

### 2.2. Chemicals

Folin-Ciocalteu reagent, gallic acid monohydrate, methanol, hydrochloric acid, ABTS (2,2-azino-bis(3-ethylbenzothiazoline-6-sulfonic acid) diammonium salt), potassium persulfate (K_2_S_2_O_8_), Trolox (6-hydroxy-2,5,7,8-tetramethylchroman-2-carboxylic acid), aluminium chloride (AlCl_3_), sodium nitrite (NaNO_2_), sodium hydroxide solution (NaOH), quercetin, sodium acetate trihydrate (CH_3_COONa·3H_2_O), glacial acetic acid (CH_3_COOH), 2,4,6-Tripyridyl-s-Triazine (TPTZ), ferric chloride (FeCl_3_), and ferrous sulphate heptahydrate (FeSO_4_·7H_2_O) were supplied from Sigma-Aldrich (Milan, Italy). Anhydrous sodium carbonate (Na_2_CO_3_) was supplied from Carlo Erba (Milan, Italy). For the preparation of the phosphate buffered saline (PBS), the following salts were used: sodium phosphate dibasic heptahydrate (HNa_2_O_4_P·7H_2_O) and sodium phosphate monobasic monohydrate (H_2_NaO_4_P·H_2_O). These were purchased from Sigma-Aldrich (Milan, Italy). All reagents were of analytical grade.

### 2.3. Cheese Manufacture

Spreadable cheese was made from pasteurised skimmed cow milk (0.1% fat) (Granarolo spa, branch office of Gioia del Colle, Italy). First, the starter composed by mesophilic aromatic culture (CHN-22 CHR Hansen) was added at 27 °C and incubated until complete acid coagulation (pH = 4.5–4.6). The formed curd was than mixed and placed into baskets lined with a cotton cloth and left to drain overnight under refrigerated conditions (4 ± 1 °C). The resulting cheese was mixed with salt (0.2% *w*/*w*) and fructo-oligosaccharide (FOS) (3% *w*/*w*) (Orafti beneo P95). After, the spreadable cheese was divided into portions of 100 g and the different by-products flours, such as broccoli (Br), artichoke (Art), tomato peel (TP), red (RGP) and white (WGP) grape pomaces, and corn bran (CB), were added at a concentration of 5% (*w*/*w*). The control cheese (CNT) without any substance was also prepared. For clarity, the cheeses supplemented with by-products are labelled as following: Br-SC (spreadable cheese enriched with broccoli), Art-SC (artichoke), TP-SC (tomato peel), RGP-SC and WGP-SC (red and white grape marc flour, respectively), and CB-SC (corn bran). All cheeses were subjected to physicochemical evaluation and sensory characterization right after their production.

### 2.4. Extraction and Determination of Total Phenolic Compounds and Total Flavonoids

The extraction of phenolic compounds from each flour and cheese sample was performed as described by Rashidinejad et al. [12] with slight modifications. Briefly, 1 g of sample was mixed with 50 mL of acidified methanol (95% MeOH containing 1% HCl) and extracted for 30 min at 50 °C in a water bath (Grant OLS200, Cambridge, UK) at 200 rpm. The mixture was cooled and filtered by cheese cloth; the residues were washed with 2 mL of the same solvent. The obtained extracts were immediately analysed. Extraction was carried out in triplicate for each sample.

Total phenolic compounds were determined in triplicate by UV-vis spectrophotometry according to the Folin-Ciocalteu method [13]. The total phenolic content was expressed as mg of gallic acid equivalents (GAEs) per g of dry weight (dw).

Total flavonoids content both in vegetable flours and in fortified cheeses was determined by the aluminium chloride colorimetric method, according to Huang et al. [14] with modifications. Each extract (0.5 mL), prepared as previously described, was mixed with 2 mL of distilled water and 150 µL of a 5% sodium nitrite (NaNO_2_) solution. After 6 min, 150 μL of a 10% aluminium chloride (AlCl_3_) solution was added and the mixture was left to rest for 6 min. Finally, 1 mL of 1 M sodium hydroxide was added and total volume was made up to 5 mL with distilled water. Then, the solutions were mixed and for each sample the absorbance was read in triplicate against blank at 415 nm. The calibration curve was prepared using quercetin as standard in the range 6.25–500 mg/L (R^2^ = 0.9985) and total amount of flavonoids was expressed in mg of quercetin equivalents (QEs) per g of dry weight (dw).

### 2.5. Antioxidant Activity

The antioxidant activity in vegetable by-products flours and cheeses was assessed using two methods: ABTS (2,2-azino-bis (3-ethylbenzothiazoline-6-sulfonic acid diammonium salt) and FRAP (ferric reducing antioxidant power) assays. The first test is based on the ability of antioxidants to interact with the radical cation ABTS^•+^ (2,2-azinobis-(3-ethylbenzothiazoline-6-sulfonic acid) inhibiting its absorption at 734 nm. The antioxidant activity was determinate according to Marinelli et al. [4] and was expressed as mg Trolox equivalents per g of dry weight. All analyses were carried out in triplicate.

The FRAP assay was carried out according to the procedure described by Benzie et al. [15] with slight modifications. The FRAP reagent was prepared by mixing 100 mL of 300 mM acetate buffer at pH = 3.6 (consisting of 3.1 g of sodium acetate trihydrate and 16 mL of acetic acid glacial per litre of buffer solution), 10 mL of 10 mM TPTZ solution (0.031 g TPTZ in 10 mL of 40 mM HCl dissolved at 50 °C) and 10 mL of 20 mM FeCl_3_ aqueous solution. The FRAP solution was prepared on the day of analysis and held at 37 °C. An amount of 3 mL of FRAP reagent was added to 200 µL of each properly diluted extract. The mixture was left to react for 30 min at 37 °C and then the absorbance was read at 593 nm. The calibration curve was obtained using as standard FeSO_4_∙7H_2_O at concentrations from 0.0125 mM to 0.6 mM (R^2^ = 0.9981). The antioxidant activity was expressed as μmols of ferrous equivalent Fe (II) per gram of dried sample. All tests were carried out in triplicate.

### 2.6. Moisture Content and pH

Moisture content was determined using a thermal balance (Sartorius, Göttingen, Germany). Five grams of homogenised sample was distributed uniformly on an aluminium pan and placed in the thermal balance set at 130 °C. For each cheese sample two replicates were measured.

The pH of the processed spreadable cheese was measured, after direct insertion of an electrode into the sample, using a previously calibrated digital pH meter (Hanna Instruments, Milan, Italy). Each measure was made twice on two different samples.

### 2.7. Sensory Characterization

The sensory characteristics of all spreadable cheese samples were evaluated by a panel of seven experienced judges (four females and three males aged between 25 years and 45 years) who were members of the Laboratory of Food Packaging. The panellists had at least several years of experience in sensory evaluation prior to this study; however, they were retrained for this study in a session of 2 h to be experienced in the products and terminology. Appropriate descriptive terms for sensory evaluation were decided during the retraining sessions. The evaluated attributes comprised six terms for flavour and aroma (overall intensity, sweetness, salty, acid, bitter, and astringent), two terms describing aftertaste (intensity and persistence), and six attributes encompassing texture and mouth feel (spreadability, fibrous, adhesiveness, solubility, graininess, juiciness). Each sensory attribute was described as reported in the publication of Niro et al. [16]. Cheese samples were rated for their spreadability on a cracker. The intensity of each attribute was evaluated on the scale from 0 to 7 (0 = not detected; 7 = very detected) [16]. The spreadable cheeses were prepared on the day of the analysis and stored at room temperature until 1 h before the evaluation.

### 2.8. Statistical Analysis

The results were compared by a one-way analysis of variance (ANOVA). A Duncan’s multiple range test, with the option of homogeneous groups (*p* < 0.05), was carried out to determine significant differences between cheese samples. The scores from the descriptive sensory analysis were used to construct a principal component analysis (PCA). STATISTICA 7.1 for Windows (StatSoft, Inc, Tulsa, OK, USA) was used for all analyses.

## 3. Results and Discussion

### 3.1. Chemical Quality

Chemical characterization of flours from by-products and of fortified spreadable cheese, in terms of total phenolic content, flavonoids, and antioxidant activity measured by ABTS and FRAP (µmol FeSO_4_·7H_2_O/g dw) assays, is shown respectively in Table 1 and Table 2.

As can be seen in Table 1, the red grape pomace powder was the by-product with the highest amount of total phenols (107.4 ± 2.08 mg GAEs/g dw), flavonoids (12.9 ± 0.39 mg QEs/g dw), and antioxidant activity (127.36 ± 2.89 mg TEs/g dw and 1886 ± 156.7 µmol FeSO_4_·7H_2_O/g dw for ABTS and FRAP, respectively) among the by-products investigated in this work, followed by the white grape pomace.

As it can be inferred from data listed in Table 2, the addition of by-products to cheese significantly increased the total phenolic content and the flavonoids, compared to the control sample, except in cheese enriched with tomato peel, probably due to the scarce source of bioactive compounds. Specifically, cheese containing white and red grape pomace recorded high phenolic content (2.74 ± 0.04 and 2.34 ± 0.15 mg GAEs/g dw, respectively) followed by broccoli (1.78 ± 0.02 mg GAEs/g dw), artichoke (1.20 ± 0.22 mg GAEs/g dw), and corn bran (0.90 ± 0.04 mg GAEs/g dw). Some unexpected results were observed, because the TPC of WGP-SC was slightly higher than that of RGP-SC. This experimental finding could be ascribed to more than one reason, for example, their stability or more simply to the method used for total phenols determination (i.e., the Folin-Ciocalteu method). As a fact, this method is not selective and therefore it can detect other reducing compounds reacting with the Folin-Ciocalteu reagent [10,12]. On the contrary, Gonzàlez-Centeno et al. [17], who studied the phenolic composition and the antioxidant activity of by-products of 10 different cultivars of *Vitis vinifera*, both white and red, observed that there was no significant difference among varieties.

From the antioxidant activity point of view, cheese fortification involved a significant increase in all the cases. The same result has been confirmed by both the ABTS and the FRAP assay. According to Alonso et al. [18], there is a positive correlation between the antioxidant activity and the total polyphenolic content of samples. In this work, best results were obtained for cheese enriched with red and white grape pomace. Other authors tried to enrich dairy products, in particular yogurt, with bioactive compounds from red grape pomace [7,10]. Tseng et Zhao [7] used wine grape pomace as a source of dietary fibre and polyphenols in yogurt and salad dressing for enhancing nutritional value and improving storability of the products. They noted a higher amount of dietary fibre and polyphenol contents and a slower lipid oxidation of samples during refrigeration storage.

Marchiani et al. [10] fortified yogurt with grape skin powder and observed a significantly higher total phenolic content and antioxidant activity than the control. Rashidinejad et al. [19] incorporated green tea extract into full-fat cheese to investigate the effect of green tea catechins on antioxidant properties and also noticed a significant increase of TPC and antioxidant activity at all concentrations.

### 3.2. Moisture Content and pH

**Figure 1:**
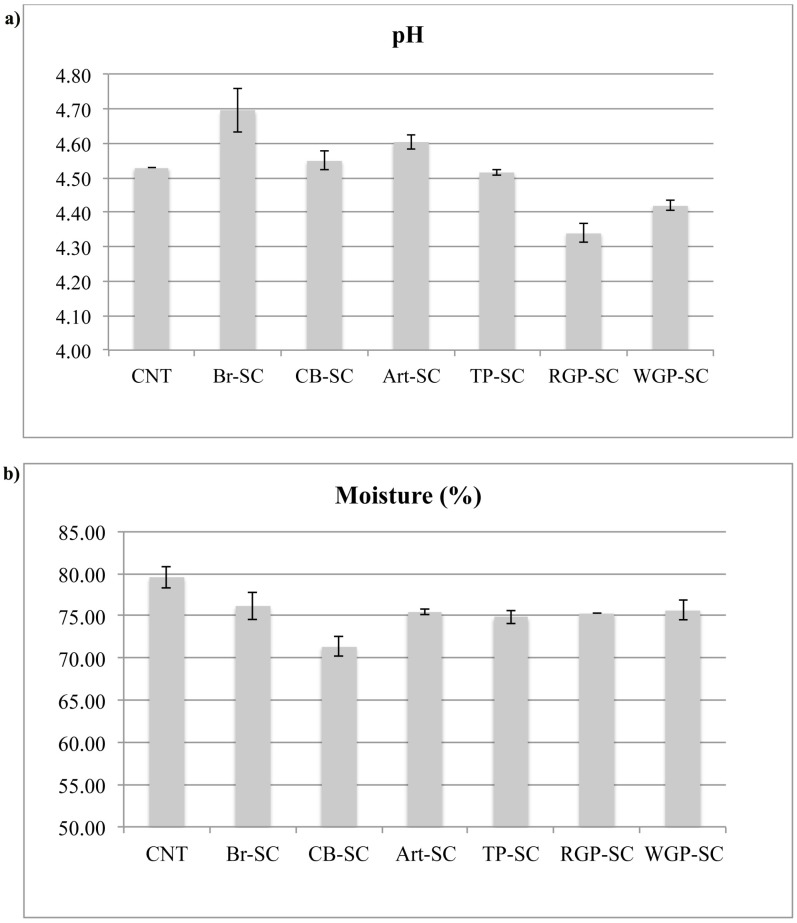
pH (**a**) and moisture content (**b**) of spreadable cheese enriched with different by-products.

Figure 1a reports the pH values of each cheese added with vegetable by-products and of the control cheese right after production. As can be observed, the pH value was 4.53 for the CNT cheese and it did not change significantly after the addition of corn bran and tomato peel by-product. Contrary, for samples with red and white grape pomace powders added, a decrease of pH from 4.53 to 4.34 and 4.42, respectively, was observed. Similar results were obtained by Tseng et al. [7], who detected a decrease of pH in yogurt immediately after the addition of wine grape pomace. For the Br-SC and Art-SC, a slight pH increase was found.

Concerning the percentage of moisture, it was observed that all tested spreadable cheese with vegetable by-products presented a moisture reduction compared to the control (Figure 1b). Probably, the addition of the by-product flours increased the total solids content in the formulation and consequently the moisture percentage decreased. In the literature, several examples confirmed a decrease of moisture content in cheese with different flours added. In particular, Safaa [20] observed that the total solid content in yogurt and soft cheese increased by increasing chia flour addition and contextually the moisture content decreased compared to control. Also, Arunkumar et al. [21] observed that the incorporation of 5%, 10%, and 15% soy flour significantly decreased the moisture of filled spreads by about 61.4%, 59.5%, and 57.7%, respectively. This redaction may be due to the relevant increase in total solid content of spreads by flour incorporation. According to El-Aziz et al. [22], the addition of food by-products increased the total solid content in cheese formulation, thus leading to a moisture decrease.

### 3.3. Sensory Quality

Ratings for flavour and textural attributes of spreadable cheeses are presented in Table 3 and Table 4, respectively.

The intensity scores for different flavours ranged among the investigated cheeses from 4 to 7 for overall intensity, acid, aftertaste intensity, and aftertaste persistence, whereas less variation (from 0 to 2) was observed for sweetness and salty. An acid taste was detected in all spreadable cheese samples, caused by the increase in acidity and by the pH decrease during the coagulation process. However, for the cheese added with red and white grape pomace powders, the acid value was higher, as confirmed by their final pH value (see Figure 1a). A pH reduction for yogurt with wine grape pomace powders added was also confirmed by Tseng and Zhao [7]. Contrary, sweetness parameter was detected only for CNT and CB-SC samples; no bitter taste was detected, except for the cheese containing the artichoke flour. This is not surprising, as it is well known that artichoke is popular for its pleasant bitter taste, which is mostly attributed to the cynarin found in the green parts of the plant [23]. Moreover, a greater astringent sensation was perceived for RGP-SC and WGP-SC samples, probably due to the presence of polyphenol substances, such as tannins, that are generally found in grape skins [24]. Regarding the textural attributes (Table 4), the CNT showed high spreadability value (score 7), while the Br-SC sample was the least spreadable among the investigated samples (score 4.5); the Art-SC, RGP-SC, and WGP-SC cheeses showed similar scores (between 5.0 and 5.5). They were found to be less spreadable with respect to the TP-SC and CB-SC samples that exhibited values of 6.0 and 6.35, respectively. The spreadability reduction may be associated to the change in moisture content and to the increase in total solid content of cheese by flour incorporation [8,10]. Fibrous, adhesiveness, and graininess attributes were also influenced by the vegetable flours; in fact, except for the CNT and the CB-SC cheese, their values ranged from 5 to 7 for all the samples. The less soluble samples like Br-SC, Art-SC, and the two grape pomace powder cheeses investigated were also the least juicy (scores from 3 to 4.5). In the literature, several examples of the effects of the water binding capacity of by-products on texture parameters and tactile sensation when incorporated into different foods (i.e., bakery, dairy, and meat products) have been reported [11,25,26,27].

**Figure 2:**
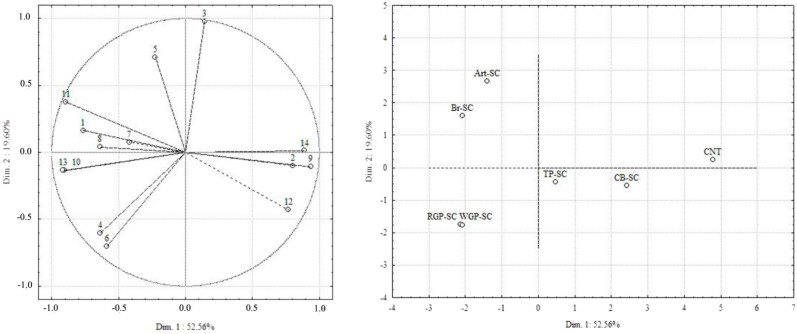
Principal components analysis of the sensory attributes of cheese (sample plot: **right**, variable plot: **left**). CNT: control spreadable cheese; CB-SC: corn bran spreadable cheese; Br-SC: broccoli spreadable cheese; WGP-SC: white grape pomace spreadable cheese; RGP-SC: red grape pomace spreadable cheese; Art-SC: artichoke spreadable cheese; TP-SC: tomato peel spreadable cheese. Attributes 1 (Overall intensity), 2 (Sweetness), 3 (Salty), 4 (Acid), 5 (Bitter), 6 (Astringent), 7 (Aftertaste Intensity), 8 (Aftertaste Persistence), 9 (Spreadability), 10 (Fibrous), 11 (Adhesiveness), 12 (Solubility), 13 (Graininess), 14 (Juiciness).

To study the variability of the sensory attributes of cheese, the principal component analysis was carried out (Figure 2).

The first dimension (Dim 1) and the second dimension (Dim 2) explained 52.56% and 19.60% of the total variability, respectively. Salty, juiciness, sweetness, spreadability, and solubility are on the right side of the plot, while all the other sensory attributes are on the left side. The first axis explains 52.56% of the total variance and separates the seven cheeses into two groups. CNT, CB-SC, and TP-SC were different from the other four cheeses on the basis of the salty, juiciness, sweetness, solubility, and spreadability sensory attributes. The other cheeses were mostly described with attributes like bitter, aftertaste, adhesiveness, overall intensity, graininess, fibrous, acid, and astringent. Within this group of four cheeses, the second axis, which explains 19.60% of the total variance, distinguished RGP-SC and WGP-SC from the other cheeses. The acid and astringent attributes on the negative part of the second axis can explain the separation of those cheeses from the other ones.

## 4. Conclusions

In this work, the influence of by-products addition on physicochemical and sensory properties of spreadable cheese was investigated. The results highlighted that the best results from a nutritional point of view were obtained for the cheese samples with red and white wine grape pomace added, followed by broccoli, artichoke, and corn bran. Generally, the addition of vegetable flour to cheese significantly increased the total phenolic content and flavonoids compared to the control sample. However, technological options could be optimized with red and white wine grape pomace added to spreadable cheese in order to increase antioxidant compounds without compromising the sensory properties.

## Figures and Tables

**Table 1:** Total phenolic content (TPC), total flavonoids content (TFC), and antioxidant activity measured by ABTS (2,2-azino-bis(3-ethylbenzothiazoline-6-sulfonic acid) diammonium salt) and FRAP (ferric reducing antioxidant power) assays of different by-products.

	TPC (mg GAEs/g dw)	TFC (mg QEs/g dw)	ABTS (mg TEs/g dw)	FRAP (µMol FeSO_4_·7H_2_O/g dw)
Broccoli by-products	14.59 ± 0.14 ^b^	5.55 ± 0.16 ^c^	23.70 ± 1.29 ^d^	212.40 ± 12.60 ^b^
Corn Bran	5.01 ± 0.08 ^a^	3.71 ± 0.02 ^b^	7.73 ± 0.30 ^b^	64.50 ± 2.68 ^a^
Artichokes by-products	21.15 ± 0.24 ^c^	9.83 ± 0.14 ^e^	16.76 ± 0.16 ^c^	267.20 ± 2.29 ^b^
Tomato peel	4.90 ± 0.48 ^a^	2.21 ± 0.02 ^a^	4.21 ± 0.27 ^a^	23.30 ± 0.63 ^a^
Red grape pomace	107.40 ± 2.08 ^e^	12.99 ± 0.39 ^f^	127.36 ± 2.89 ^f^	1886.00 ± 156.70 ^d^
White grape pomace	90.51 ± 0.36 ^d^	8.84 ± 0.10 ^d^	110.13 ± 2.90 ^e^	1619.00 ± 85.30 ^c^

^a–f^: Data in columns with different superscripts are significantly different (*p* < 0.05). Results are expressed as means ± Standard Deviation for *n* = 3.

**Table 2:** Total phenolic content (TPC), total flavonoids content (TFC), and antioxidant activity measured by ABTS and FRAP assays of spreadable cheese enriched with 5% by-products.

	TPC (mg GAEs/g dw)	TFC (mg QEs/g dw)	ABTS (mg TEs/g dw)	FRAP (µMol FeSO_4_·7H_2_O/g dw)
CNT	0.66 ± 0.06 ^a^	0.47 ± 0.00 ^a^	0.96 ± 0.08 ^a^	1.52 ± 0.05 ^a^
Br-SC	1.78 ± 0.02 ^d^	0.79 ± 0.07 ^c^	2.09 ± 0.06 ^d^	6.65 ± 0.20 ^d^
CB-SC	0.90 ± 0.04 ^b^	0.49 ± 0.03 ^a^	1.71 ± 0.09 ^c^	4.53 ± 0.47 ^c^
Art-SC	1.20 ± 0.22 ^c^	0.06 ± 0.03 ^b^	1.71 ± 0.20 ^c^	4.74 ± 0.13 ^c^
TP-SC	0.72 ± 0.02 ^a^	0.47 ± 0.00 ^a^	1.51 ± 0.07 ^b^	2.58 ± 0.12 ^b^
RGP-SC	2.34 ± 0.15 ^e^	0.86 ± 0.08 ^d^	3.95 ± 0.19 ^e^	26.17 ± 0.72 ^e^
WGP-SC	2.74 ± 0.04 ^f^	0.89 ± 0.03 ^d^	4.00 ± 0.06 ^e^	26.45 ± 0.25 ^e^

CNT: control spreadable cheese; Br-SC: broccoli spreadable cheese; CB-SC: corn bran spreadable cheese; Art-SC: artichoke spreadable cheese; TP-SC: tomato peel spreadable cheese; RGP-SC: red grape pomace spreadable cheese; WGP-SC: white grape pomace spreadable cheese. ^a–f^: Data in columns with different superscripts are significantly different (*p* < 0.05). Results are expressed as means ± Standard Deviation for *n* = 3.

**Table 3:** Ratings for flavour attributes of seven spreadable cheeses investigated.

	Flavour Attributes (0–7)							
Samples	Overall Intensity	Sweetness	Salty	Acid	Bitter	Astringent	Aftertaste Intensity	Aftertaste Persistence
CNT	6.0 ± 0.3 ^b^	1.2 ± 0.4 ^a^	1.0 ± 0.0 ^a.b^	4.6 ± 0.4 ^b^	n.d.	n.d.	6.0 ± 0.0 ^b^	6.0 ± 0.0 ^c^
Br-SC	7.0 ± 0.0 ^c^	n.d.	1.4 ± 0.4 ^b^	5.1 ± 0.2 ^b^	n.d.	1.2 ± 0.4 ^a^	7.0 ± 0.2 ^c^	6.7 ± 0.4 ^d^
CB-SC	5.6 ± 0.3 ^a^	2.0 ± 0.0 ^b^	0.7 ± 0.3 ^a^	4.0 ± 0.2 ^a^	n.d.	2.0 ± 0.0 ^b^	5.6 ± 0.3 ^b^	5.4 ± 0.2 ^b^
Art-SC	6.9 ± 0.2 ^c^	n.d.	1.6 ± 0.4 ^b^	4.9 ± 0.4 ^b^	6.0 ± 0.0	0.8 ± 0.4 ^a^	6.6 ± 0.4 ^c^	6.2 ± 0.3 ^c^
TP-SC	5.5 ± 0.3 ^a^	n.d.	0.5 ± 0.5 ^a^	6.0 ± 0.0 ^c^	n.d.	0.9 ± 0.4 ^a^	5.0 ± 0.3 ^a^	4.1 ± 0.2 ^a^
RGP-SC	7.0 ± 0.0 ^c^	n.d.	n.d.	7.0 ± 0.0 ^d^	n.d.	3.0 ± 0.0 ^c^	7.0 ± 0.0 ^d^	7.0 ± 0.0 ^d^
WGP-SC	6.9 ± 0.2 ^c^	n.d.	n.d.	6.9 ± 0.2 ^d^	n.d.	3.0 ± 0.3 ^c^	7.0 ± 0.2 ^d^	6.2 ± 0.3 ^c^

^a–d^： Data in each column with different superscripts are significantly different (*p* < 0.05). Results are expressed as means ± Standard Deviation. n.d.: not detected; CNT: control spreadable cheese; Br-SC: broccoli spreadable cheese; CB-SC: corn bran spreadable cheese; Art-SC: artichoke spreadable cheese; TP-SC: tomato peel spreadable cheese; RGP-SC: red grape pomace spreadable cheese; WGP-SC: white grape pomace spreadable cheese.

**Table 4:** Rating for textural attributes of spreadable cheeses investigated.

	Textural Attributes (0–7)					
Samples	Spreadability	Fibrous	Adhesiveness	Graininess	Solubility	Juiciness
CNT	7.0 ± 0.0 ^d^	n.d.	4.0 ± 0.0 ^a^	n.d.	6.0 ± 0.3 ^d^	6.0 ± 0.0 ^c^
Br-SC	4.5 ± 0.4 ^a^	6.0 ± 0.0 ^b^	5.5 ± 0.3 ^d^	5.1 ± 0.2 ^b^	3.0 ± 0.3 ^a^	3.9 ± 0.2 ^a^
CB-SC	6.3 ± 0.2 ^c^	4.5 ± 0.4 ^a^	4.5 ± 0.4 ^b^	3.9 ± 0.2 ^a^	5.0 ± 0.3 ^c^	4.5 ± 0.4 ^b^
Art-SC	5.5 ± 0.3 ^b^	6.3 ± 0.2 ^c^	5.5 ± 0.0 ^d^	5.5 ± 0.0 ^c^	3.9 ± 0.2 ^b^	4.0 ± 0.0 ^a^
TP-SC	6.0 ± 0.0 ^c^	6.5 ± 0.0 ^c^	5.1 ± 0.2 ^c^	5.1 ± 0.2 ^b^	4.0 ± 0.0 ^b^	4.0 ± 0.3 ^a^
RGP-SC	5.3 ± 0.2 ^b^	7.0 ± 0.2 ^d^	5.0 ± 0.0 ^c^	6.0 ± 0.0 ^d^	4.5 ± 0.4 ^c^	4.0 ± 0.3 ^a^
WGP-SC	5.1 ± 0.2 ^b^	7.0 ± 0.0 ^d^	5.1 ± 0.2 ^c^	6.0 ± 0.3 ^d^	4.6 ± 0.4 ^c^	4.0 ± 0.3 ^a^

^a–d^： Data in each column with different superscripts are significantly different (*p* < 0.05). Results are expressed as means ± Standard Deviation. n.d.: not detected; CNT: control spreadable cheese; Br-SC: broccoli spreadable cheese; CB-SC: corn bran spreadable cheese; Art-SC: artichoke spreadable cheese; TP-SC: tomato peel spreadable cheese; RGP-SC: red grape pomace spreadable cheese; WGP-SC: white grape pomace spreadable cheese.
